# Validity and Reliability of the Malay Versions of Bloating Severity (BSQ-M) and Quality of Life (BLQoL-M) Questionnaires

**DOI:** 10.3390/ijerph18052487

**Published:** 2021-03-03

**Authors:** Nurzulaikha Mahd-Ab.lah, Yee Cheng Kueh, Garry Kuan, Fatan Hamamah Yahaya, Mung Seong Wong, Nor Aslina Abd Samat, Nurhazwani Hamid, Nurhayati Mohamad Nor, William E. Whitehead, Syed Ismail Thiwan, Yeong Yeh Lee

**Affiliations:** 1 Biostatistics and Research Methodology Unit, School of Medical Sciences, Universiti Sains Malaysia, Kubang Kerian 16150, Kelantan, Malaysia; ngahpc@yahoo.com; 2 Exercise and Sport Science, School of Health Sciences, Universiti Sains Malaysia, Kubang Kerian 16150, Kelantan, Malaysia; 3 Department of Life Sciences, Brunel University, London UB8 3PH, UK; 4 School of Distance Education, Universiti Sains Malaysia, USM, Penang 11800, Pulau Pinang, Malaysia; hamamah@usm.my; 5 Medical Department, School of Medical Sciences, Universiti Sains Malaysia, Kubang Kerian 16150, Kelantan, Malaysia; mswong@usm.my (M.S.W.); aslina.samat@gmail.com (N.A.A.S.); azamhazwani2528@gmail.com (N.H.); hayati.nmn@gmail.com (N.M.N.); 6 Division of Gastroenterology and Hepatology CB 7080, Chapel Hill Department of Medicine, University of North Carolina, 4112 Bioinformatics Bldg, Chapel Hill, NC 27599-7080, USA; william_whitehead@med.unc.edu; 7 Department of Medicine, Eastern Virginia Medical School, Norfolk, VA 23507, USA; syedthiwan@gmail.com; 8 Gut Research Group, Faculty of Medicine, National University of Malaysia, Kuala Lumpur 43600, Selangor, Malaysia

**Keywords:** abdominal bloating, questionnaire, severity, intention, quality of life, factor analysis

## Abstract

Abdominal bloating (AB) is a prevalent and bothersome symptom, but there are no specific measures for severity and quality of life (QoL) other than the Bloating Severity Questionnaire (BSQ) and Bloating Quality of Life (BLQoL). We aimed to translate the BSQ and BLQoL into the Malay language and to validate them using exploratory factor analysis (EFA) and confirmatory factor analysis (CFA) approaches. The 12-item BSQ has two components, seven-item severity in general (SevGen) and five-item severity in the past 24 h (Sev24), and BLQOL has five items. Translation to the Malay language (BSQ-M and BLQoL-M) was performed using standard forward and backward processes. EFA followed by CFA were performed in participants with AB due to functional bowel disorders, with the purpose of examining the validity and reliability of the questionnaires translated into Malay. After EFA with 152 participants, all the items of BSQ-M remained in the model. Total variance extracted was 53.26% for BSQ-M and 58.79% for BLQoL-M. The internal consistency based on Cronbach’s alpha values was 0.52 for SevGen, 0.86 for Sev24, and 0.81 for BLQoL-M. After performing CFA with another 323 participants, the final measurement model for BSQ-M and BLQoL-M fit the data well in terms of several fit indices (BSQ-M: root mean square error of approximation (RMSEA) = 0.050, Comparative Fit Index (CFI) = 0.966, Tucker–Lewis Fit Index (TLI) = 0.956, and standardized root mean squared residual (SRMR) = 0.051; BLQoL-M: RMSEA = 0.071, CFI = 0.985, TLI = 0.962, SRMR = 0.021). The composite reliability for BSQ-M and BLQoL-M were satisfactory (SevGen = 0.83, Sev24 = 0.89, BLQoL = 0.80). The intraclass correlation (ICC) results showed excellent stability for BSQ-M and BLQoL-M, ranging from 0.74 to 0.93. The Malay language versions of BSQ-M and BLQoL-M are valid and reliable instruments for measuring the severity and QoL of AB for the Asian population with functional bowel disorders.

## 1. Introduction

Abdominal bloating (AB) is a common complaint during clinical consultations [1,2], and 1 in 10 Americans report bloating even without heavy meals [2,3]. While considered to be a mere nuisance to some individuals, bloating may be a sign of serious bowel disease and hepatobiliary or pancreatic disease [4,5,6]. Bloating also affects the general measure of well-being and disease-specific quality of life (QoL) [6]. Although bloating can be stand-alone, there are often other accompanying symptoms that increase the severity of the disease [7,8,9,10,11,12]. The ROME IV criteria for functional abdominal bloating/distension stated that it falls under bowel disorders, with the description of recurrent episodes on average at least one day per week and the symptoms predominating over other symptoms (that may co-exist with mild abdominal pain and minor bowel disorder) and that are insufficient to classify with other diagnosis with onset of 6 months and are present during the last 3 months [1]. The management of AB can be complex due to the above reasons [13,14,15,16,17].

Rao [17] reported that 44% of 1602 patients with irritable bowel syndrome–constipation (IBS-C) had severe fullness and bloating, 32% had severe discomfort, 23% had severe pain, and 22% had severe cramping. Exact bloating mechanisms are unknown but may involve the following: inability of the abdominal wall muscles to adapt to changes of intraabdominal volumes, abnormal production of intestinal gas, dysbiosis or presence of small intestinal bacterial overgrowth (SIBO), altered gut motility and impaired gas handling, and visceral hypersensitivity. While the presence of AB may be confounded by factors such as psychological factors, hormonal factors, smoking, and obesity [11,18,19], the severity of AB may be associated with fluid retention; IBS [11], particularly diarrhea-predominant IBS [18]; food intolerance; and drugs (e.g., high-dose aspirin) [19].

There are only general measures of severity and QoL of AB, for example, IBS Severity Score (IBSSS) [20], Short Form Health Survey (SF-36) [21], Functional Bowel Disorder Severity Index (FBDSI) [22,23], IBS-QoL [24,25], Gastrointestinal Quality of Life Index (GIQLI) [6], EUroQoL-5 dimension (EQ5D) [26,27], Gastroparesis Cardinal Symptom Index (GCSI) [5], Rating Form of IBD Patient Concerns [28], EuroQoL [29], Health-Related Quality of Life (HRQoL) [30], IBS-SS [31], The Internal Gas Questionnaire [32], Short Form of Inflammatory Bowel Disease Questionnaire (SIBDQ) [33], self-administered lower bowel symptoms [34], Inflammatory Bowel Disease Questionnaire [35,36], and Bowel Disease Questionnaire [37,38]. Moreover, owing to differences in language and perception of illness, these scales may not be valid outside of their original language and across different cultures. In addition, most of the existing tools focus on evaluating the relationship of AB with other bowel diseases, and there are no specific self-report measures for severity and QoL of AB. Specific tools for severity and QoL of AB are important for comparing the effectiveness and safety of treatment trials.

Thiwan et al. [39,40,41,42] first developed the BSQ and BLQoL using a literature review, previous assessment methods, and internet-based adult focus groups with AB. From a preliminary eight-question BSQ, following pilot testing in 58 patients, the authors further expanded it to 12 questions to accommodate 2 subscales, i.e., a seven-item general severity (SevGen) scale and a five-item severity in the past 24 h (Sev24) scale. The BLQoL is a four-item questionnaire, including bloating interference with work, intimate relationships, hobbies, and social activities, which was later finalized to five items with one item added to capture emotion-related effect. With psychometric testing, both BSQ subscales showed good internal consistency and correlated with BLQoL impairment [42]. In a subsequent study to assess the responsiveness of BSQ employing a cross-over trial design, 19 patients with AB due to lactose intolerance were first randomized to group 1: lactose 240 mL twice daily for one week, followed by three-day washout, then lactose-free for one week, or group 2: lactose free for the first week, washout, and then lactose 240 mL twice daily for one week, before cross-over [41]. Participants were asked to complete the Sev24 daily and SevGen weekly. It was found that Sev24 was highly sensitive to changes in bloating but not for SevGen (probably due to short duration of intervention). 

Thus, the aims of current study were to translate the original BSQ and BLQoL into the Malay language, and to examine the reliability and validity of these instruments before they can be used clinically for addressing patients with a complaint of AB. 

## 2. Materials and Methods

### 2.1. Study Design, Participants, and Sampling Method

Using cross-sectional study design, we consecutively sampled patients with a complaint of AB who were receiving treatment at Hospital Universiti Sains Malaysia (HUSM). All participants met the eligibility criteria of the study, including the following: participants with complaint of bloating (satisfied the ROME IV items of bloating, and or participants who had at least experienced 1 episode of bloating on the basis of response to the question “have you ever experience bloating?” and/or using a pictogram (approval obtained from the ROME Foundation)); 18 years of age and above; able to read, write, or understand the Malay language; available at the time of data collection; and consented to participation. Participants were not restricted to disorders of IBS or functional bloating. Participants were not restricted to disorders of IBS or functional bloating. However, functional bloating was the minimum requirement for joining the study. Therefore, the two functional bloating items were used in both the exploratory factor analysis (EFA) and confirmatory factor analysis (CFA) studies. Then, additional items on IBS and general diagnosis item (“has your doctor diagnosed you with any listed medical problem?”) were used to examine other types of gastrointestinal issues. Exclusion criteria were participants who had a history of abdominal surgeries or taking bloatedness-related drugs. Non-probability sampling method (purposive sampling) was applied when recruiting the participants. An additional 40 participants, using similar eligibility criteria, were approached for test–retest analysis with the purpose of examining the stability of the questionnaire in detecting the intended outcome of interest.

### 2.2. Questionnaire Translation

The BSQ and BLQoL questionnaires were translated from English to Malay language using a standardized procedure of questionnaire translation outlined by Brislin [43,44,45,46,47,48]. Firstly, the original English questionnaires were translated into the Malay language by 2 independent bilingual translators. Secondly, the translated Malay version questionnaires were then back-translated to the English language by another independent bilingual translator. Thirdly, the 3 different translators and the researcher met and discussed both the forward and backward versions to finalize the final draft. Fourthly, 5 experts with good experience in health psychology, psychometrics, bloating, and questionnaire development reviewed the preliminary questionnaires for their contents and cultural appropriateness. The semi-final questionnaires were pre-tested on 30 participants with AB for clarity and comprehension. They were encouraged to raise any issues related to the wording and the presentation of the questionnaires. The results of the pre-tests were good, and no modification was needed. The participants commented that they understood the written instruction and the items listed in the questionnaires. The presentation of the questionnaires was acceptable, and the wordings were comprehended by the participants. The final Malay version of BSQ and BLQoL then proceeded for validation, which consisted of exploratory and confirmatory analyses.

### 2.3. Measures

#### 2.3.1. Demographic Information

The questionnaire set included items related to complaint of bloating and socio-demographic information, such as sex, age, ethnicity, religion, height, weight, living area (either rural, meaning village/countryside, or urban, meaning town/city) and history of other medical and surgical illnesses, which were also used to understand the characteristics of the participant. In addition to complaint to treating clinicians, AB was defined and diagnosed on the basis of the ROME IV questionnaires of functional bloating and additional items on IBS subtypes. 

#### 2.3.2. BSQ and BLQoL

The original English version of BSQ was developed by Thiwan et al. [43] from Chapel Hill and consists of two components, i.e., seven-item SevGen and five-item Sev24. In terms of the work of Thiwan et al., we found an an abbreviated version of a three-item Sev24, with similar internal consistency with the five-item Sev24, but for the current study, only the original five-item Sev24 was used [40]. Likewise, the original English version of BLQoL was developed by Thiwan et al. [42], and consisted of 5 items, i.e., bloating interference with work, intimate relationships, hobbies, social activities and the additional emotion item. Responses for BSQ and BLQoL were in Likert scale points format. Responses for BSQ were in a multiple-choice answer format on different degrees of effect towards individuals on the basis of intensity, frequency, and severity (less severe to more severe; range 1 to 4, 5, 6, 7 or 8 varied by items). For SevGen, items 1, 3, 4, 5, and 6 measured a 1–5 scale, item 2 measured a 1–4 scale, and item 7 measured a 1–7 scale. For Sev24, all items measured a 1–5 scale, except for item 5 (which measured 1–8 scale). Responses for BLQoL were in a seven-point Likert scale on different degrees of impact towards individual ranging from 1 = “never/not related to me” to 7 = “always”. Higher scores in BSQ indicate worse severity and higher score in BLQoL indicate higher impact towards QoL [42]. Both subscales of BSQ had good internal consistency (Cronbach’s alpha values for SevGen and Sev24 were 0.76 and 0.85, respectively) [42].

### 2.4. Procedure

The study received approval from the Universiti Sains Malaysia’s Human Research Ethics Committee (USMKK/PPP/JEPEM/17010012) and conformed to guidelines of the International Declaration of Helsinki. Written informed consent was obtained from each participant. The estimated time to complete all questionnaires was approximately 10–15 min. The completion and returning of the questionnaire together with the signed consent form indicated consented participation. The participants were reminded that the participation was voluntary, and that they could withdraw at any time during the study. The researcher who was involved in the data collection was not a healthcare provider or their clinician. During the data collection, the researcher ensured the participants that their participation or withdrawal in this study would not have any impact on their treatment in the hospital facilities.

There were 520 potential participants screened, and eventually 510 participants fulfilled the eligibility criteria. Of 510 participants, 160 were approached for the exploratory study and 350 for the confirmatory study. Among all who returned the questionnaires, 475 (152 for exploratory factor analysis (EFA) and 323 for confirmatory factor analysis (CFA)) were complete and usable for subsequent data analysis. For test–retest reliability and stability of the questionnaires, we invited an additional 40 people from the 323 participants for CFA to complete the questionnaires again after 14 days.

### 2.5. Statistical Analysis

Data were entered into SPSS version 26 (SPSS Inc., Chicago, IL, USA) for descriptive analysis, EFA, and internal consistency analysis. Mplus version 8 was used for CFA. Data were presented as mean and standard deviations for numerical variables and frequency and percentages for categorical variables, unless mentioned otherwise. 

For EFA, principal axis factoring with Promax rotation (kappa 4) was used to explore the domains. The threshold cut off point of 0.4 and higher was used to differentiate good factor loading items within the domains [49,50,51]. Then, internal consistency test based on the Cronbach’s alpha was used to assess reliability of each component in BSQ and BLQoL. The internal consistency based on Cronbach’s alpha with threshold value of 0.60 is generally recommended [49,50], however, values above 0.50 was still considered acceptable in some of the literature [51,52]. The final EFA model was then tested with CFA. 

For CFA, the initial hypothesized measurement model was identified through the EFA results. Items with factor loadings of less than 0.40 or standardized residual value of more than 4.0 were considered problematic and iteratively removed. To further improve the measurement model, we inspected the modification index (MI) from the Mplus output and added items’ error covariance if necessary. The re-specification of the measurement model was carried out by researchers with adequate theoretical support. 

Several fit indices were used to determine model fitness, and their threshold values are as follows: standardized root mean squared residual (SRMR) lower than 0.06, the root mean square error of approximation (RMSEA) less than 0.08, Tucker–Lewis Fit Index (TLI) above 0.920, and Comparative Fit Index (CFI) above 0.920 [46,47,48].

Composite reliability (CR) based on Raykov’s method was used to determine the reliability of each factor in the final measurement model [53,54]. The acceptable value for CR was 0.60 and above [46,47,48,55,56,57]. Average variance extracted (AVE) of the final measurement model was also computed and the acceptable value was 0.50 and above [55]. Pearson correlation test was used as an added analysis to check the discriminant validity between the domains [47,52,53]. For test–retest analysis, the intraclass correlation (ICC) was used to examine the stability of BSQ and BLQoL. A value of ICC more than 0.70 was considered excellent [58,59].

## 3. Results

Of the total 475 (152 EFA + 323 CFA) participants, the overall mean age was 29.39 years old (SD = 13.13, ranged from 18 to 93), more than half were females (*n* = 305, 64.2%), and 54.1% (*n* = 257) were from the rural population. There were 16.8% (*n* = 80) and 8.2% (*n* = 39) who reported other symptoms and other diseases, respectively. All were found to have the presence of functional bloating through EFA. For CFA, additional ROME IV items related to IBS revealed that there were IBS bloating-related cases of IBS-C, IBS-D, and IBS-M with percentages of 23.2%, 11.8%, and 14.6%. The items for general diagnosis by a physician shared by the respondents found that there were 1.5%, 0.3%, 1.5%, 1.5%, 1.2%, and 0.6% cases for functional dyspepsia, chronic constipation, chronic diarrhea, diabetes, celiac disease, and diverticulitis, respectively. The remaining 43.8% still satisfied functional bloating criteria.

### 3.1. Exploratory Factor Analysis

Data for each item in BSQ-M and BLQoL-M are normally distributed on the basis of a histogram. The questionnaires were analyzed separately on the basis of the two main components, Severity and QoL. The initial principal axis factoring analysis of all 12 items in BSQ-M and 5 items in BLQoL-M indicated sampling adequacy. Thus, we found a reliable estimate from our current models with computed Kaplan–Meier–Olkin (KMO) values of 0.798 and 0.772 for BSQ-M and BLQoL-M, respectively. For Bartlett’s test of sphericity, both scales were significant (*p* < 0.001). The items were run with EFA to explore the domain for BSQ-M and BLQoL-M. The next step was proceeded with fixing the number of factors to two for BSQ-M and one for BLQoL-M, which were parallel to the model proposed by the original author. 

All of the subscale scores were normally distributed. For severity (BSQ-M), the mean and SD for each item are summarized in Table 1. The internal consistency for severity 24 h domain (factor 1) in BSQ-M was good, with a Cronbach’s alpha (α) of 0.86. For severity general (factor 2), the Cronbach’s alpha was 0.52 and was considered acceptable for further testing. All items exceeded the cut point of 0.4 for good factor loading, except for severity general items 1 and 2. As such, the problematic items were retained for further analysis to maintain the content as developed by the original author. Principal axis factoring EFA with Promax rotation produced two subscale scores: scale 1 (seven items, eigenvalue = 4.32), measuring symptom severity for 24 h, accounting for 36.01% of the variance in BSQ-M score, and scale 2, measuring symptom severity for general (five items, eigenvalue = 2.07), accounting for 17.25% of variance in score. 

For the QoL part (BLQoL-M), the means and SDs for each item are summarized in Table 2. The internal consistency of one domain of QoL was good (Cronbach’s α = 0.81). All items exceeded the cut point of 0.4 suggested good factor loadings. As such, all items were retained as the original five-item scale. Principle axis factoring EFA with Promax rotation produced one subscale scores for all five items with an eigenvalue of 2.94 and measured symptom impact to quality of life that accounted for 58.79% of the variance in BLQoL-M score. 

### 3.2. Confirmatory Factor Analysis

To further understand the constructs evaluated by the BSQ-M and BLQoL-M, we performed secondary analyses by using CFA. The preliminary model from EFA result was used in the initial measurement model in CFA. The fit indices were examined, and amendments were made accordingly to achieve the best model representing the constructs. For severity, the fit indices failed to reach the cut point threshold value for fit model, and suitable amendments were made. When we considered the factor loading, standardized residual variances, and MI, one item (SEVG2 from BSQ-M) was removed from the model with the original author’s permission. Thus, as shown in Table 3, both of the constructs had good final fit indices.

Mean scores for each item in each scale for component severity are presented in Table 4. The severity part in BSQ-M demonstrated good construct validity and reliability on the basis of the factor loading all higher than 0.40), AVE more than 0.50, and the CR more than 0.60.

In terms of quality of life component (BLQoL-M), the mean scores for each item are presented in Table 5. The BLQoL-M showed good construct validity in terms of the factor loadings that were all higher than 0.40, with AVE more than 0.50. BLQoL-M also showed good reliability through CR with value of 0.796.

**Figure 1:**
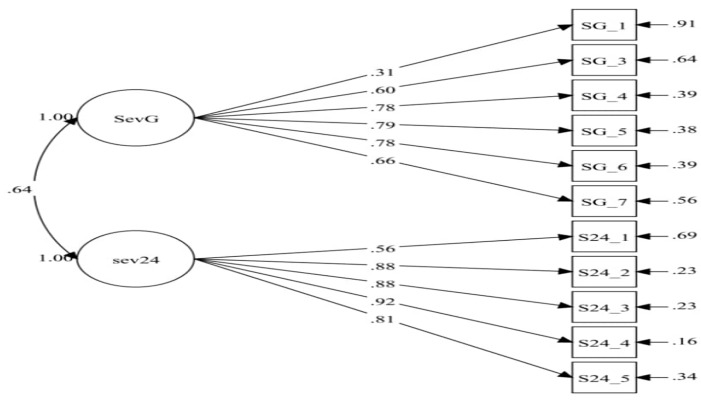
Model of severity in the Bloating Severity Questionnaire in the Malay language (BSQ-M) consisting of two domains (sevg = severity general and sev24 hrs = severity 24 h). SG_1 = SEVG1, SG_3 = SEVG3, SG_4 = SEVG4, SG_5 = SEVG5, SG_6 = SEVG6, SG_7 = SEVG7, S24_1 = SEV241, S24_2 = SEV242, S24_3 = SEV243, S24_4 = SEV244, S24_5 = SEV245.

**Figure 2:**
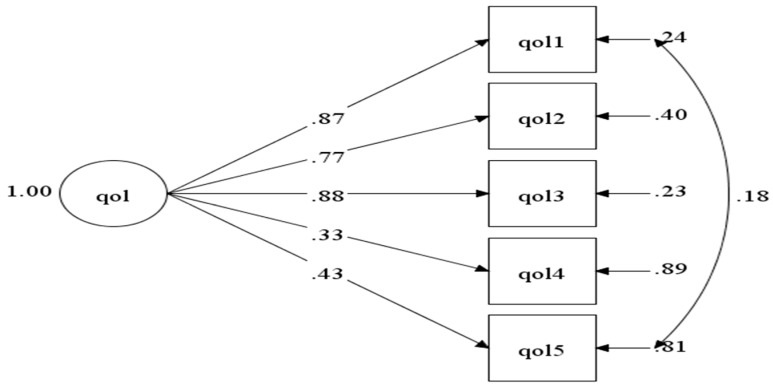
Model of quality of life domain in BLQoL-M.

Below is the simulation of the model inside the BSQ-M (Figure 1) and BLQoL-M (Figure 2) in CFA.

### 3.3. Discriminant Validity

Pearson’s correlation test was performed to add on the evidence discriminant validity. The positive moderate correlation between SevGen and Sev24 (*R* = 0.44, *p* < 0.001) indicated that each question contributed uniquely to the BSQ-M. The correlations between factors were below 0.85, indicating that the discriminant validity of the BSQ-M was satisfied.

### 3.4. The Internal Consistency (ICC)

The mean ICC for test–retest reliability after two weeks showed excellent results for all the three domains (all were more than 0.75) among 40 participants. The ICC reported for each domain were 0.74 (0.51, 0.86) for severity 24 h, 0.87 (0.75, 0.93) for severity general, and 0.93 (0.84, 0.97) for QoL. This indicated that the two scales (BSQ-M and BLQoL-M) were stable over two time periods. 

## 4. Discussion

In the present study, the newly translated BSQ-M and BLQoL-M questionnaires are shown to be valid and reliable tools for specific measurements of severity and QoL of AB. EFA was first utilized to explore items if they remain in the same domains as proposed by the original authors. Furthermore, the original questionnaire was validated in a different population from ours. In this regard, the Promax rotation principal axis factoring analysis was a suitable approach to explore the structure of the data for problematic items [60]. Then, the CFA was utilized to reconfirm the items in each domain after the EFA, and we found two items were problematic from the SevGen scale. One item (SEVG2 = “On days that you have bloating, how often does it usually happen?” “*Pada hari anda mengalami kembung perut, berapa kerapkah ia biasa berlaku?*”) was rather general and measured a broad area, and thus we decided to remove the item; this was agreed by the original authors. In addition, the mean total score was used to explain the level of severity and QoL instead of each item score, and while total score allowed for an overall picture of severity, but this could be biased since different subscales have different sensitivity to AB, as shown in the subsequent lactose study [41].

Our study differs from the previous study because we focused on individuals who had experienced AB in their lifetime, regardless of severity, regardless of the symptoms occurring once or several times in their lifetime, and without concerning the disease nature of AB [55]. Since we were validating the instruments, a broad range of cases with symptom of AB should have ideally been included, however, in this study, we utilized largely benign functional gastrointestinal disorders since this indication was the largest population with AB in our clinical practice. From the present study, all items had factor loadings above the acceptable threshold value of 0.4, except for item 4 in BLQoL-M. This was expected, as item 4 measures the impact of AB specifically on married participants, yet the majority of our participants were single. Thus, it was decided that this item would not be excluded even though it was statistically problematic.

The present study demonstrated good reliability in terms of the Cronbach’s alpha, ICC, and CR values. All of these indices strengthened the worth of these measures for use in practice and for further studies. Some of the related questionnaires were the intestinal gas questionnaire [32], the bowel disease questionnaire [37,38], IBS-SS [31], the GIQLI score [6], and others [30,34,35]. However, these questionnaires do not explicitly measure the severity and QoL of AB [23,24,25,26,27,28,29,30,31,32,33,34,35,36,37,38]. Therefore, this study provided new insight into the health outcome measurement tools that are valid and reliable to measure the severity of AB and QoL in clinical practice for people with AB symptoms.

Measurement of the severity and impact to QoL were lacking for the essential components in the complaint of AB and were also an important factor to account for when assessing the treatment effectiveness among other bowel disorders. Thus, the BSQ-M and BLQoL-M should be significant tools to consider in future studies in measuring the impact of individual treatment outcomes explicitly for AB symptoms.

There are some limitations to this study. First, the data all come from a single center and the study site is the referral center for AB, mostly due to functional bowel disorders. Second, these were self-reported questionnaires and hence prone to response bias, but the researchers had repeatedly reminded the participants to be as honest and as accurate as possible. Third, the complaint of AB was based on a number of tools because of the heterogeneity of AB, including culture and language differences between ethnic groups. Fourth, the marital status of study population was predominantly single, and thus some items were rendered problematic during EFA and CFA.

## 5. Conclusions

The present study presents the Malay version of the BSQ and BLQoL for use in research and clinical work assessing the severity and impact towards QoL, especially for work and social interaction among AB patients. These findings demonstrate that the BSQ-M and BLQoL-M are two short, simple-to-administer, valid self-report measures of severity and QoL for the Malaysian population using Malay as the lingua franca. 

## Figures and Tables

**Table 1:** Descriptive statistics for severity general and severity 24 h (*n* = 150).

No. Abbreviated Item Content	Mean	*SD*	Factor Loading	
			Factor 1	Factor 2
SEVG1 (“In the last month, how many days total would estimate you have had any type of bloating?” “Pada bulan lepas, berapa jumlah anggaran hari anda mengalami apa-apa jenis kembung perut?”)	1.91	1.16		−0.246
SEVG2 * (“On days that you have bloating, how often does it usually happen?” “Pada hari anda mengalami kembung perut, berapa kerapkah ia biasa berlaku?”)	2.14	1.07		0.176
SEVG3 (“How long does the bloating typically last each time it is present?” “Berapa lama selalunya kembung perut berlarutan pada setiap kali ia berlaku?”)	2.07	0.80		0.422
SEVG4 (“How severe is your bloating typically?” “Berapa terukkah kembung perut anda selalunya?”)	2.14	0.80		0.536
SEVG5 (“How often do you have pain along with the bloating?” “Berapa kerap anda berasa sakit dengan kembung perut?”)	2.22	0.70		0.790
SEVG6 (“When you have pain with your bloating, how severe is your pain typically?” “Apabila anda mengalami kesakitan semasa kembung perut, betapa terukkah sakit anda selalunya?”)	2.09	0.65		0.693
SEVG7 (“How often do you have discomfort other than pain along with the bloating?” “Berapa kerapkah anda berasa tidak selesa selain daripada kesakitan semasa kembung perut?”)	2.26	0.75		0.554
SEV241 * (“How often have you had bloating over the past 24 h?” “Berapa kerapkah anda mengalami kembung perut sejak 24 jam yang lepas?”)	4.03	1.17	0.655	
SEV242 (“How severe was your bloating, in terms of its effects on you, in the past 24 h?” “Berapa terukkah kesan kembung perut ke atas anda dalam masa 24 jam yang lepas?”)	1.59	0.63	0.904	
SEV243 (“How much pain that was related to the bloating did you have in the past 24 h?” “Sejauh mana kesakitan yang berkaitan dengan kembung perut yang anda rasai pada 24 jam yang lepas?”)	1.52	0.68	0.803	
SEV244 (“How much discomfort other than pain did you have related to your bloating in the past 24 h?” “Sejauh mana ketidakselesaan selain daripada sakit yang anda alami berkaitan dengan kembung perut pada 24 jam yang lepas?”)	1.63	0.69	0.931	
SEV245 (“What proportion of your total waking time did you have any bloating in the past 24 h (adding together the length of all bloating episodes you had during that time)?” “Apakah pecahan jumlah masa jaga yang mana anda mengalami kembung perut pada 24 jam lalu (dengan menambah tempoh masa kembung perut yang anda alami pada masa itu?”)	1.97	1.19	0.738	
Eigenvalue			4.32	2.07
Variance explained (%)			36.01	17.25
Cumulative variance (%)			36.01	53.26
Cronbach’s alpha			0.86	0.52

Factor 1, symptom severity for general; factor 2, symptom-severity for 24 h. SD = standard deviation. Note: * means reverse item.

**Table 2:** Descriptive statistics for quality of life domain (*n* = 150).

No. Abbreviated Item Content	Mean	*SD*	Factor Loading
QOL1 (“When you are bloated, how often does the bloating limit or restrict your ability to work or attend school?” “Apabila perut anda berasa kembung, berapa kerapkah ia mengehadkan keupayaan anda untuk bekerja atau belajar?”)	2.24	1.00	0.818
QOL2 (“When you are bloated, how often does the bloating limit or restrict your ability to participate in social activities?” “Apabila perut anda berasa kembung, berapa kerapkah ia mengehadkan keupayaan anda untuk mengambil bahagian dalam aktiviti sosial?”)	2.10	1.01	0.878
QOL3 (“When you are bloated, how often does the bloating limit or restrict your ability to enjoy hobbies or recreational activities?” “Apabila perut anda berasa kembung, berapa kerapkah ia mengehadkan anda daripada menikmati hobi atau aktiviti rekreasi?”)	1.91	0.97	0.842
QOL4 (“When you are bloated, how often does the bloating limit or restrict your ability to enjoy intimate relationships?” “Apabila perut anda berasa kembung, berapa kerapkah ia mengehadkan anda daripada menikmati hubungan intim?”)	1.72	1.11	0.448
QOL5 (“When you are bloated, how often does the bloating affect you emotionally?” ”Apabila perut anda berasa kembung, berapa kerapkah ia menggangu emosi anda?”)	2.26	0.70	0.458
Eigenvalue			2.94
Variance explained (%)			58.79
Cumulative variance (%)			58.79
Cronbach’s alpha			0.81

**Table 3:** Summary for Bloating Quality of Life in the Malay language (BLQoL)-M model fit indices (*n* = 323).

Path Model *	RMSEA (90% CI)	CFI	TLI	SRMR
Severity				
Model 1	0.129 (0.116, 0.143)	0.776	0.721	0.084
Model 2	0.050 (0.031, 0.067)	0.966	0.956	0.051
QoL				
Model 1	0.079 (0.036, 0.127)	0.975	0.949	0.021
Model 2	0.071 (0.019, 00.125)	0.985	0.962	0.021

* The models were separately tested for validity. Model 2 for severity summarized after SEVG2 removed. Model 2 for QoL summarized after adding correlated items residual QOL5 with QOL1.

**Table 4:** Factor loading, convergent reliability, and convergent validity of the confirmatory factor analysis for the severity domain (*n* = 323).

Constructs/Items	Mean	*SD*	λ	AVE	CR
Severity General				0.550	0.831
SEVG1	1.46	0.64	0.307		
SEVG2 *	2.06	0.85	-		
SEVG3	1.79	0.75	0.604		
SEVG4	2.27	0.82	0.784		
SEVG5	2.37	0.73	0.788		
SEVG6	2.31	0.72	0.783		
SEVG7	2.41	0.91	0.662		
Severity 24 h				0.673	0.889
SEV241 *	2.45	1.31	0.557		
SEV242	1.87	0.91	0.877		
SEV243	1.76	0.86	0.877		
SEV244	1.78	0.88	0.919		
SEV245	2.12	1.19	0.813		

Note: λ = standardized factor loading, CR = construct reliability; all factor loadings were statically significant at *p* < 0.050. Note: * means reverse item.

**Table 5:** Factor loading, convergent reliability, and convergent validity of the confirmatory factor analysis for the quality of life domain (*n* = 323).

Constructs/Items	Mean	*SD*	λ	AVE	CR
Quality of Life				0.543	0.796
QOL1	2.19	0.91	0.871		
QOL2	2.17	0.90	0.774		
QOL3	2.22	0.98	0.878		
QOL4	3.30	1.61	0.325		
QoL5	1.44	1.01	0.433		

## Data Availability

The data presented in this study are available on request from the corresponding author. The data are not publicly available due to privacy and ethical restrictions.
